# Experimental Inoculation of Egyptian Fruit Bats (*Rousettus aegyptiacus*) with Ebola Virus

**DOI:** 10.3390/v8020029

**Published:** 2016-01-22

**Authors:** Janusz T. Paweska, Nadia Storm, Antoinette A. Grobbelaar, Wanda Markotter, Alan Kemp, Petrus Jansen van Vuren

**Affiliations:** 1 Centre for Emerging and Zoonotic Diseases, National Institute for Communicable Diseases of the National Health Laboratory Service, Sandringham 2131, South Africa; nadias@nicd.ac.za (N.S.), antoinetteg@nicd.ac.za (A.A.G.); alank@nicd.ac.za (A.K.); petrusv@nicd.ac.za (P.J.V.); 2 School of Pathology, Faculty of Health Sciences, University of the Witwatersrand, Johannesburg 2193, South Africa; 3 Department of Microbiology and Plant Pathology, University of Pretoria, Pretoria 0028, South Africa; Wanda.Markotter@up.ac.za

**Keywords:** Egyptian fruit bat, experimental inoculation, Ebola virus, seroconversion, tissue tropism, shedding

## Abstract

Colonized Egyptian fruit bats (*Rousettus aegyptiacus*), originating in South Africa, were inoculated subcutaneously with Ebola virus (EBOV). No overt signs of morbidity, mortality, or gross lesions were noted. Bats seroconverted by Day 10–16 post inoculation (p.i.), with the highest mean anti-EBOV IgG level on Day 28 p.i. EBOV RNA was detected in blood from one bat. In 16 other tissues tested, viral RNA distribution was limited and at very low levels. No seroconversion could be demonstrated in any of the control bats up to 28 days after in-contact exposure to subcutaneously-inoculated bats. The control bats were subsequently inoculated intraperitoneally, and intramuscularly with the same dose of EBOV. No mortality, morbidity or gross pathology was observed in these bats. Kinetics of immune response was similar to that in subcutaneously-inoculated bats. Viral RNA was more widely disseminated to multiple tissues and detectable in a higher proportion of individuals, but consistently at very low levels. Irrespective of the route of inoculation, no virus was isolated from tissues which tested positive for EBOV RNA. Viral RNA was not detected in oral, nasal, ocular, vaginal, penile and rectal swabs from any of the experimental groups.

## 1. Introduction

The family *Filoviridae* comprises the *Ebolavirus*, *Marburgvirus*, and *Cuevavirus* genera. The genus *Ebolavirus* includes five species, each represented by a single virus member: *Sudan ebolavirus* (Sudan virus, SUDV), *Zaire ebolavirus* (Ebola virus, EBOV), *Bundibugyo ebolavirus* (Bundibugyo virus, BDBV), *Taï Forest ebolavirus* (Taï Forest virus, TAFV), and *Reston ebolavirus* (Reston virus, RESTV). With the exception of RESTV and Lloviu virus (LLOV, species *Lloviucuevavirus*), all known filoviruses cause severe hemorrhagic fever in humans [1]. The unprecedented scale of Ebola virus disease (EVD) outbreaks in West Africa in 2013–2015 caused by EBOV [2,3] represent a dramatic expansion of case numbers and introduction of this highly lethal virus into new geographic areas, a frightening account for the prospect of EBOV turning into a formidable public health threat globally. Despite the increase in EVD outbreaks in recent years [4], the natural reservoirs of filoviruses remain elusive [5,6].

Circumstantial evidence for an association between ebolaviruses and bats was reported in the first SUDV outbreak in 1976 [7] and in the TAFV outbreak in chimpanzees in Ivory Coast in 1994 [8]. In the first experimental filovirus infection study of bats, conducted in 1996 by Swanepoel *et al.* [9], wild-caught Angolan free-tailed bats (*Mops condylurus*), little free-tailed bats (*Chaerephon pumilus*) and Wahlberg’s epauletted fruit bats (*Epomophorus wahlbergi*) sustained replication of EBOV following subcutaneous inoculation, in the absence of clinical disease, thus supporting the potential role of bats in harboring filoviruses. The first detection of EBOV-specific IgG and viral RNA was reported in 2005 in wild-caught fruit bats of three species (*Hypsignathus monstrosus*, *Epomops franqueti*, and *Myonycteris torquata*) sampled in areas of Gabon and the Republic of the Congo affected by EVD outbreaks [10]. Several subsequent field studies have confirmed anti-EBOV IgG in the same species [11] and also implicated a number of other fruit bat species, including *Rousettus aegyptiacus*, in Ghana, Gabon, and the Republic of Congo [11,12,13,14]. Antibodies against RESTV were reported in *Rousettus spp.* bats in the Philippines [15] and Bangladesh [16], and in insectivorous and fruit bat species in China [17]. Although very circumstantial, *Mops condylurus* bats, which replicated EBOV upon experimental infection previously [9], were seen close to the putative index case of the West African EVD outbreak [18]. Antibodies to EBOV and RESTV were recently reported in the migratory straw-colored fruit bat, *Eidolon helvum* [19]. To date, however, attempts to isolate ebolaviruses from both fruit and insectivorous bat species have been unsuccessful [6].

Egyptian fruit bats were consistently shown to be a source of Marburg virus transmissions to humans [20,21,22]. In recent years marburgviruses were repeatedly isolated from *Rousettus aegyptiacus* bats in Uganda [21,22,23]. Experimental infections of *R. aegyptiacus* with Marburg virus resulted in virus replication in blood and multiple tissues [24,25,26], and oral shedding [26] without evidence of overt disease. However, an attempt to transmit Marburg virus horizontally from experimentally infected to susceptible in-contact *R. aegyptiacus* bats, was unsuccessful [25].

Antibodies against EBOV and RESTV have been detected in wild *Rousettus* spp. bats in Africa [11,12], the Philippines [15], Bangladesh [16], and China [17] and EBOV replication was demonstrated in a *R. aegyptiacus*-derived cell line [27]. Based on these results it has been postulated that *Rousettus* spp. bats might act as reservoir hosts of ebolaviruses. However, captive-bred *R. a*egyptiacus bats originating from Uganda were recently shown to be refractory to experimental inoculation with ebolaviruses [28]

In this study we expanded the EBOV experimental inoculation work in *R. aegyptiacus* bats by Jones *et al.* [28] using colonized *R. aegyptiacus* bats originating in South Africa in a larger euthanasia study. We also report on the first attempt to transmit EBOV horizontally from subcutaneously (s.c.) inoculated to in-contact control bats, and on the serological responses, tissue tropism, viral shedding, and clinical and gross pathological effects in *R. aegyptiacus* bats exposed to EBOV by three different inoculation routes.

## 2. Materials and Methods

### 2.1. Ethics Statement

This work was done in agreement with the recommendations of the South African National Standards for the Care and Use of Animals for Scientific Purposes (SANS 10386:2008) and with permission to conduct research in terms of Section 20 of the Animal Disease Act issued by the Department of Agriculture, Forestry and Fisheries of the Republic of South Africa (permission no. 12/11/1/1/13). The procedures for establishing the *R. aegyptiacus* colony and inoculating bats with the Ebola virus were approved by the National Health Laboratory Service Animal Ethics Committee (clearance numbers. AEC 136/12and AEC 141/13, respectively). Blood was collected under anesthesia and exsanguinated animals were dissected as described in Paweska *et al.* [24]. All efforts were made to minimize distress.

### 2.2. Experimental Animals

The source of *R. aegyptiacus* bats and the trapping procedures were the same as described previously for the experimental infection study with Marburg virus (MARV) [24]. Wild caught bats were transported to a biosafety level 3 (BSL3) animal quarantine facility where they were held, fed and tested prior to moving to a flight cage for colonization as previously described [24]. All work with infectious EBOV and inoculated animals was conducted at the Centre for Emerging and Zoonotic Diseases, National Institute for Communicable Diseases, National Health Laboratory Service, Sandringham, South Africa in a biological safety level 4 (BSL4) laboratory. Husbandry procedures and environmental conditions were identical to those described in Paweska *et al.* [24]. Animals were acclimated to the BSL4 environment for one week before the experiment started.

### 2.3. Virus

The SPU 220/96 isolate of EBOV (4th passage in Vero cells) used to inoculate bats was isolated from the serum of a nurse who contracted a fatal infection from a Gabonese physician admitted to a private hospital in South Africa in 1996 [29].

### 2.4. Experimental Inoculations

Thirty-six EBOV-seronegative bats, aged 4–24 months, were used during the first stage of the experiment. Twenty-four bats were inoculated subcutaneously (s.c.) with 100 µL of tissue culture supernatant containing 10^5.0^ 50% tissue culture infectious doses/mL (TCID_50_/mL) of EBOV, and 12 control bats were mock-inoculated s.c. with 100 µL of Eagles Minimal Essential Medium (EMEM). Subcutaneous inoculation was administered into the loose skin over the shoulders. Animals were split up into 6 cages, each containing 4 EBOV-infected and 2 in-contact control bats. Sampling of blood and tissues was carried out as previously described [24]. The sampling framework from s.c. inoculated bats is given in Table 1. Blood was collected from in-contact control bats on Days 0, 14, 21 and 28 post inoculation (p.i.).

**Table 1:** Sampling schedule, quantitative reverse transcription PCR (Q-RT-PCR) and virus isolation results in blood and tissues of Egyptian fruit bats (*Rousettus aegyptiacus*) inoculated subcutaneously with Ebola virus.

Sample Type, Bat ID, Sex and Number of Bats Tested Post Mortem	Days after Inoculation					
	3 (*n* = 4)^a^	5 (*n* = 3)	7 (*n* = 5)	10 (*n* = 3)	21 (*n* = 4)	37 (*n* = 5)
	17^b^ (M), 103 (F), 106 (F), 110 (M)	20 (M), 81E (F), 452 (F)	02D (F), 100 (M), 105 (F), 109 (M), 111 (M)	112 (F) 47B (F), 426 (F)	1 (F), 33 (M), 91B (F), 113 (F)	5 (F), 31(F), 104 (F), A63 (F), E0F (M)
Blood	1/13^c^; 106 38.21^d^,VI^−^	0/11	0/5	0/3	n.t.	n.t.
Liver	0/4	0/3	1/5; 02D 39.34, VI^−^	0/3	0/4	0/5
Spleen	0/4	0/3	0/5	0/3	0/4	0/5
Kidney	0/4	0/3	0/5	0/3	0/4	0/5
Lung	0/4	0/3	1/5; 02D 36.31, VI^−^	0/3	0/4	0/5
Small intestine	0/4	0/3	0/5	n.s.	0/4	0/5
Large intestine	0/4	0/3	0/5	n.s.	0/4	0/5
Stomach	0/4	0/3	0/5	n.s.	0/4	0/5
Reproductive^e,f^organs	0/4	0/3	0/5	n.s.	0/4	0/5
Bladder	0/4	0/3	0/5	n.s.	0/4	0/5
Rectum	0/4	0/3	0/5	n.s.	0/4	0/5
Heart	0/4	0/3	0/5	n.s.	0/4	0/5
Skin	0/4	0/3	0/5	n.s.	0/4	0/5
Muscle	0/4	0/3	0/5	n.s.	0/4	0/5
Salivary glands	0/4	0/3	0/5	n.s.	0/4	0/5
Conjunctiva	0/4	0/3	0/5	n.s.	0/4	0/5
Brain^g^	0/4	0/3	0/5	n.s.	0/4	0/5

^a^ Number of bats tested post mortem; ^b^ Bat identification number; ^c^ Number of bats with positive samples/total number of bats tested post mortem and alive; ^d^ Q-RT-PCR *Ct* value; M—Male; F—Female; VI^−^—Negative virus isolation; n.s.—Not sampled; n.t.—Not tested; ^e^ Pooled sample of testicles, epididymides; ^f^ Pooled sample of ovaries, uterine horns and uterus; ^g^ Pooled sample of cortex, brain stem, basal ganglia, cerebellum.

During the second stage of the experiment, 11 bats that remained seronegative after 28 days in-contact exposure to EBOV-infected bats were inoculated intraperitoneally (i.p.; *n* = 6), and intramuscularly (i.m; *n* = 5) with the same dose of EBOV as for s.c. inoculation. Intraperitoneal inoculation was administered in the lower right quadrant of the animal’s abdomen, close to the midline above the genitalia. Intramuscular inoculation was given into caudal thigh muscle. Sampling of blood and other tissues from i.p. and i.m. inoculated bats is given in Table 2. Oral, nasal, vaginal, penile and rectal swabs were collected from all the EBOV-inoculated groups at regular intervals (Table 3). Animals were anaesthetized prior to inoculation and specimen collection as previously described [24]. Bats were monitored daily for the development of clinical signs as well as for food intake. Post mortem tissues were processed as previously described [24].

**Table 2:** Sampling schedule, quantitative reverse transcription PCR (Q-RT-PCR) and virus isolation results in blood and tissues of Egyptian fruit bats (*Rousettus aegyptiacus*) inoculated intraperitoneally (i.p.) and intramuscularly (i.m.) with Ebola virus.

Sample Type, bat ID, Sex and Number of Bats Tested Post Mortem	Day after i.p. and i.m. Inoculation		
	5 (*n* = 4)^a^	7 (*n* = 2)	16 (*n* = 5)
	I.P.: 16^b^ (F), 101 (M)	I.P.: 102 (F)	I.P.: 37 (M), 759 (F), 944 (F)
	I.M.: 21 (F), 107 (F)	I.M.: 108 (M)	I.M.: 15 (M), 15C (M)
Blood	I.P.: 0/2 ^c^; I.M.: 0/2	I.P.: 1/1 (102: 35.08, VI^−^)	I.P.: 0/3
		I.M.: 1/1 (108: 36.8, VI^−^)	I.M.: 1/2 (15: 37.89, VI^−^)
Liver	I.P.: 0/2; I.M.: 0/2	I.P.: 0/1	I.P.: 0/3; I.M.: 0/2
		I.M.: 1/1 (108: 40.0, VI^−^)	
Spleen	I.P.: 0/2; I.M.: 0/2	I.P.: 1/1 (102: 40.0, VI^−^)	I.P.: 0/3
		I.M.: 0/1	I.M.: 1/2 (15: 35.88, VI^−^)
Kidney	I.P.: 0/2; I.M.: 0/2	I.P.: 1/1 (102: 36.66, VI^−^)	I.P.: 0/3; I.M.: 0/2
		I.M.: 0/1	
Lung	I.P.: 0/2; I.M.: 0/2	I.P.: 0/1; I.M.: 0/1	I.P.: 0/3; I.M.: 0/2
Small intestine	I.P.: 0/2; I.M.: 0/2	I.P.: 0/1; I.M.: 0/1	I.P.: 0/3; I.M.: 0/2
Large intestine	I.P.: 0/2	I.P.: 0/1; I.M.: 0/1	I.P.: 0/3; I.M.: 0/2
	I.M.: 1/2 (107: 39.17^d^, VI^−^)		
Stomach	I.P.: 2/2 (101: 39.68; 16: 39.4, VI^−^)	I.P.: 0/1; I.M.: 0/1	I.P.: 0/3; I.M.: 0/2
	I.M.: 1/2 (107: 38.93, VI^−^)		
Reproductive organs^e,f^	I.P.: 0/2; I.M.: 0/2	I.P.: 0/1; I.M.: 0/1	I.P.: 0/3; I.M.: 0/2
Bladder	I.P.: 0/2; I.M.: 0/2	I.P.: 0/1; I.M.: 0/1	I.P.: 0/3; I.M.: 0/2
Rectum	I.P.: 0/2; I.M.: 0/2	I.P.: 0/1; I.M.: 0/1	I.P.: 0/3; I.M.: 0/2
Heart	I.P.: 0/2; I.M.: 0/2	I.P.: 0/1; I.M.: 0/1	I.P.: 0/3; I.M.: 0/2
Skin	I.P.: 1/2 (101: 39.57, VI^−^)	I.P.: 0/1; I.M.: 0/1	I.P.: 0/3; I.M.: 0/2
	I.M.: 0/2		
Muscle	I.P.: 0/2; I.M.: 0/2	I.P.: 0/1; I.M.: 0/1	I.P.: 0/3; I.M.: 0/2
Salivary glands	I.P.: 0/2; I.M.: 0/2	I.P. 0/1; I.M.: 0/1	I.P. 0/3; I.M.: 0/2
Conjunctiva	I.P.: 0/2; I.M.: 0/2	I.P.: 0/1; I.M.: 0/1	I.P.: 0/3; I.M.: 0/2
Brain^g^	I.P.: 0/2; I.M.: 0/2	I.P.: 0/1; I.M.: 0/1	I.P.: 0/3; I.M.: 0/2

^a^ Number of bats tested post mortem; ^b^ Bat identification number; ^c^Number of bats with positive samples/total number of bats tested post mortem and alive; ^d^ Q-RT-PCR *Ct* value; M—Male; F—Female; VI^−^—Negative virus isolation; ^e^ Pooled sample of testicles, epididymides; ^f^ Pooled sample of ovaries, uterine horns and uterus; ^g^ Pooled sample of cortex, brain stem, basal ganglia, cerebellum.

**Table 3:** Quantitative reverse transcription PCR results in swabs collected from bats inoculated by different routes with Ebola virus.

Type of Swab	Subcutaneous d.p.i.^a^				Intraperitoneal or Intramuscular d.p.i.	
	3	5	7	10	5	7
Oral	0/13 ^b^	0/11	0/3	0/3	0/4	0/2
Nasal	0/13	0/11	0/3	0/3	0/4	0/2
Ocular	0/13	0/11	0/3	0/3	0/4	0/2
Vaginal/penile	0/13	0/11	0/3	0/3	0/4	0/2
Rectal	0/13	0/11	0/3	0/3	0/4	0/2

^a^ Days post inoculation; ^b^ Number of bats with positive samples/number bats tested.

### 2.5. Serology

Sera from virus and mock inoculated animals were tested by an indirect IgG ELISA, as described in Paweska *et al.* [24], modified to use recombinant EBOV glycoprotein (GP) (Integrated BioTherapeutics, Gaithersburg, MD) antigen (3.1 mg/mL) diluted 1:6000 in PBS pH 7.2, and positive control serum from the Wahlberg’s epauletted fruit bat (*Epomophorus wahlbergi*) experimentally infected with EBOV [9]. The mean OD ELISA readings were converted to a percentage of positive (PP) control serum as previously described [30].

### 2.6. Real-Time Quantitative RT-PCR (Q-RT-PCR)

Processing of blood, tissues, and swabs for quantitative reverse-transcription PCR (Q-RT-PCR) targeting the L polymerase gene of EBOV [31] was performed as previously described [24]. Primers Filo A2.4 and Filo B were used according to the protocol published by Panning *et al.* [31] except for using a 0.1µM concentration of the FAMEBOg probe. Samples with *C_t_* values ≤40 were regarded as positive.

A quantification standard was produced by cloning the EBOV L gene PCR target region into a pCRII-TOPO expression vector (Invitrogen) as described in Paweska *et al.* [24]. Other internal controls incorporated an extraction control and a template-free control. Copy numbers of EBOV RNA detected per reaction volume were converted to copy numbers per milliliter of plasma or gram of tissue. For converting RNA copy numbers detected in samples to a TCID_50_, a logarithmic titration curve using stock EBOV SPU 220/96 isolate was generated as previously described [24].

### 2.7. Virus Isolation

Processing of specimens for virus isolation was performed as described in Paweska *et al.* [24]. Virus isolation was attempted on all Q-RT-PCR positive blood and tissues. Cultures were tested for EBOV replication by Q-RT-PCR using tissue culture fluids collected at the time of first visible cytopathic effect (CPE) or at 14 days p.i.

### 2.8. Statistical Analysis

The cut-off value for the ELISA was determined as two times the mean plus 3-fold standard deviations (SD) of ELISA PP values recorded in 36 experimental animals before inoculation.

## 3. Results

The ELISA cut-off value was determined as 36.7 PP. Q-RT-PCR performed on RNA extracts from log_10_ dilutions of the challenge EBOV in EMEM yielded a linear correlation between RNA copy numbers and TCID_50_ (*R^2^* = 0.9819). One TCID_50_/mL was equivalent to 5 × 10^3^ EBOV viral RNA per mL of blood or g of tissue, corresponding to Q-RT-PCR Ct value of 35. Extracts of RNA from log_10_ dilutions of EBOV below one TCID_50_/mL were all negative (*C*t values >40, results not shown).

All bats remained clinically well and maintained normal food uptake; no gross pathology was detected. None of the bats sustained apparent skin or mucous wounds or injury for the duration of the experiments.

**Figure 1:**
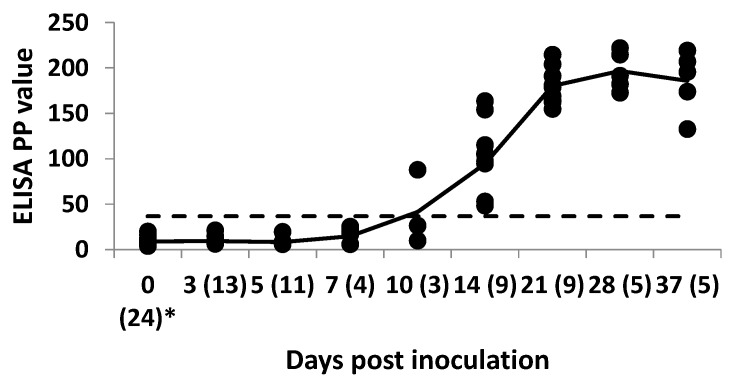
Individual (●) and mean (solid line) IgG levels in 24 Egyptian fruit bats inoculated subcutaneously with Ebola virus (EBOV) in a serial euthanasia study. Results for anti-EBOV IgG antibody by enzyme-linked immunosorbent assay are shown as percent positivity (PP) of ELISA positive internal control serum. ELISA cut-off value of 36.7 PP (dashed line). The values in brackets indicate the number of individual bats sampled on a particular day post inoculation.

**Figure 2:**
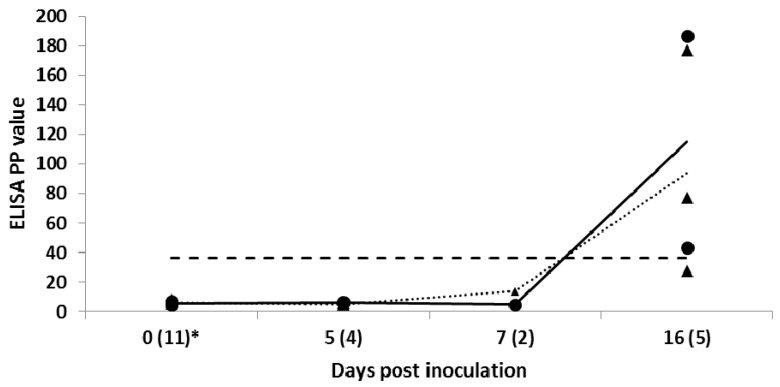
Individual and mean IgG levels in Egyptian fruit bats inoculated intraperitoneally (▲, dotted line; *n* = 5) and intramuscularly (●, solid line; *n* = 6) with Ebola virus (EBOV) in a serial euthanasia study. Results for anti-EBOV IgG antibody by enzyme-linked immunosorbent assay are shown as percent positivity (PP) of ELISA positive internal control serum. ELISA cut-off value of 36.7 PP (dashed line). The values in brackets indicate the number of individual batssampled on a particular day post inoculation.

In EBOV-inoculated bats, the first seroconversion was detected in one out of three bats tested on Day 10 p.i. On Day 14 p.i, all s.c. inoculated animals had seroconverted. The highest mean IgG antibody level was recorded on Day 28 p.i., followed by a slight decrease by Day 37 after s.c. inoculation (Figure 1). Seroconversion was not detected in any of the 12 in-contact bats. In i.p. and i.m. inoculated bats, seroconversion was detected in all but one i.p. inoculated bat on Day 16 p.i. (Figure 2).

Except for one bat (No. 106) on Day 3 p.i., the blood of the remaining s.c. inoculated bats tested on Days 3, 5, 7 and 10 p.i. were all qRT-PCR negative (Table 1). EBOV RNA was detected in the blood of i.p. and i.m. inoculated bats on Day 7 p.i., and one out of two i.m. inoculated bats on Day 16 p.i. (Table 2).

Of the 16 other tissues from s.c. inoculated bats, EBOV RNA was detected in the liver and the lung in a single bat on Day 7 p.i. (Table 1). In the i.p. inoculated bats, EBOV was detected in the stomach of two bats and the skin of one bat on Day 5 p.i., and in the spleen and kidney of one bat on Day 7 p.i. In i.m. inoculated bats, EBOV RNA was detected in the stomach of one of two bats on Day 5 p.i., in the liver of one bat on Day 7 p.i., and in the spleen in one of two bats on Day 16 p.i. The *C*t values of the Q-RT-PCR ranged from 35.08 to 38.21 in blood, and from 35.88 to 40 in the other tissues, an equivalent of less than 1 TCID_50_/mL of plasma, or 1 TCID50/g of tissue. Irrespective of the inoculation route, all EBOV RNA-positive blood samples and tissues were negative by virus isolation (Table 1 and Table 2). Viral RNA was not detected in any of oral, nasal, vaginal, penile or rectal swabs collected from EBOV-inoculated bats on Days 3–10 p.i. (Table 3).

## 4. Discussion

Jones *et al.* [28] reported the experimental infection of groups of four captive-bred, juvenile Egyptian fruit bats inoculated s.c. with each of the five known ebolaviruses. Irrespective of the virus used, no bat became viremic, there was no evidence for viral shedding, and tissue distribution of viral RNA was limited in all but SUDV-inoculated bats. In a follow-up experiment [28] involving 15 bats inoculated s.c. with SUDV, viral RNA was detected in 8 out of 10 different tissues, but in the absence of detectable viremia or viral shedding. The only tissues in which SUDV levels were high enough to suggest possible viral replication were the inoculation site, at Days 3 and 6 p.i., and the spleen in one bat, on Day3 p.i. However, of the 15 bats, viral RNA was detected only in one tissue in eight individuals (skin or axillary lymph node), and in one bat in two tissues (skin and liver). These results seem to indicate notable differences in individual response to s.c. inoculation with SUDV. In contrast, s.c inoculation of *R. aegyptiacus* with MARV consistently results in systemic infection, including relatively long viremia, viral replication in multiple tissues, and detection of viral RNA in oral and rectal swabs [24,25,26,28].

We expanded the pilot EBOV experimental work by Jones *et al.* [28] using colonized *R. aegyptiacus* bats originating in South Africa in a larger serial euthanasia study. This is the first experimental infection study attempting in-contact transmission of EBOV in bats, as well as comparing tissue tropism, viral shedding, and clinical and gross pathological effects in *R. aegyptiacus* exposed to EBOV by s.c, i.p. and i.m. routes. We studied the infection kinetics in 16 different tissues from Days 3–37 and in the blood from Days 3–10 after s.c. inoculation with EBOV. In our study, low levels of EBOV RNA was detected in 2 out of 24 bats (0.83%) on Days 3 (blood) and 7 (liver and lung) p.i., with negative Q-RT-PCR results in all tissues sampled on Days 21 and 37 p.i. There was no evidence for viral shedding as demonstrated by negative Q-RT-PCR results in different swabs and lack of horizontal transmission of the virus from EBOV-inoculated to in-contact control bats. Although in our study urine samples were not collected, during handling of animals they consistently urinated, thus vaginal and penile swabs tested were inherently contaminated with urine. None of the immunologically privileged sites tested (brain, testis) were PCR-positive. Our findings strongly suggest that s.c. inoculation of *R. aegyptiacus* with EBOV does not result in acute or chronic infection. It is unlikely that EBOV can spread among caged bats in the absence of detectable viral replication and shedding. However, it should be taken into account that horizontal virus transmission may be intermittent and slower, and subjected to external factors, including stresses, pregnancy, estrous, and starvation.

It appears that compared to s.c. inoculation, exposure of *R. aegyptiacus* to EBOV by the i.p. and i.m. routes results in wider tissue distribution of viral RNA and detection in a higher proportion of inoculated bats. EBOV RNA was detected in two out of six (33.3%) i.p. inoculated, and in two out of five (40%) i.m. inoculated bats. In this context, i.m inoculation mimics a possible natural exposure route for virus transmission, e.g., via bite. Irrespective of the route of inoculation and the type of tissues tested in this study, the recorded *C*t values were consistently very high (>35), indicating extremely poor replication of EBOV in the major tissues of *R. aegyptiacus* bats. This has been substantiated further by failure to isolate virus from Q-RT-PCR-positive blood and tissue samples. A direct correlation between Marburg virus RNA levels (viral load) in *R. aegyptiacus* determined by Q-RT-PCR and the ability to isolate virus was also shown by Towner *et al.* [21]. Although replication of EBOV in *R. aegyptiacus* below the detection limit in Vero cells seems to be unlikely, passaging of blood and tissue homogenates which tested low positive by Q-RT-PCR is worth considering in future studies.

In the absence of any convincing laboratory evidence for EBOV replication, the easily detectable and relatively strong anti-EBOV IgG responses in bats from all experimental groups are rather intriguing. In the study by Jones *et al.* [28], terminated at Day 10 p.i., no seroconversion was detected against any of the five ebolaviruses. However, in bats inoculated with SUDV in a serial euthanasia study terminated Day 15 p.i., one out of six surviving bats seroconverted on Day10 p.i., and two out of three had seroconverted on Day 15 p.i. Our serological results are similar, but we also demonstrated that bats inoculated s.c. with EBOV can maintain high antibody levels up to Day 37 p.i. The kinetics of anti-EBOV IgG responses in i.p. and i.m. inoculated bats seem to be similar. Our serological results and those from Jones *et al.* [28] could be explained by an initial immune response to the viral antigen present in the inoculums and/or to viral antigens of low-level replicating virus at the inoculation site. SUDV viral RNA loads were shown to be highly suggestive of viral replication at the inoculation site where viral antigen was also demonstrated in small numbers of macrophages in the deep subcutis. In one instance the SUDV antigen was also present in an axillary lymph node [28]. We could detect the EBOV RNA in the skin of only one bat but in our study skin specimens were taken from the ventral thorax, not from the dorsal inoculation site.

Interestingly, the *in vivo* findings by Jones *et al.* [28] and our study contrast with the *in vitro* study by Krähling *et al.* [27] who showed replication of EBOV in an Egyptian fruit bat-derived cell line similar to the replication kinetics observed in Vero E6 cells [27], and morphological changes similar to those noted in EBOV-infected cell cultures originating from monkeys and humans. Despite efficient replication of EBOV in the immortalized fetal cells from the Egyptian fruit bat, the virus did not replicate efficiently *in vivo*. Thus, caution should be used when inferring host status of certain species based on *in vitro* results.

Our larger serial euthanasia study confirms earlier results in smaller experimental group of bats [28] that *R. aegyptiacus* is refractory to infection with EBOV. Experimental inoculation was, however, sufficient to induce easily detectable antibody. A similar situation might occur in nature. Thus, serological findings in experimentally inoculated bats are important for the interpretation of epidemiological significance in wild-caught bats. Firstly, it demonstrates that it is impossible to identify bat species as filovirus reservoirs entirely based on seropositivity. On the other hand, the identification of EBOV-seropositive Egyptian rousettes in Gabon [11,12] might indicate their contact with another bat species or other hosts which are infected with and shedding the virus. Communal feeding by different fruit bat species or other mammals on the same fruit-bearing tree might represent a possible route of incidental exposure of Egyptian fruit bats to the actual reservoir species. Natural exposure of *R. aegyptiacus* to EBOV could occur during fighting with infected bats of different species or inoculation of the virus by bite of infected ectoparasites [5]. If bat ectoparasites do migrate between co-roosting or co-feeding bat species, it could explain why bats of different species test positive for anti-EBOV IgG [6].

Although reasonably low, the passage history of EBOV isolate used in this study may have influenced the outcome of our results in experimentally inoculated *R. aegyptiacus*, including the severity of infection, level of viral replication, tissue tropism, and shedding pattern. The effect of *in vitro* EBOV passage history on genetic and biological characteristics of this virus remains to be investigated.

## 5. Conclusions

In conclusion, our findings further confirm that *R. aegyptiacus* bats are unlikely to maintain and perpetuate EBOV in nature. However, natural ports of filovirus entry and the resulting viral replication and shedding patterns in *R. aegyptiacus* are unknown. In this context, the likelihood of altered EBOV infection profiles following discrete exposure routes warrant further investigations. While *R. aegyptiacus* might not be a suitable investigational model for studying ebolavirus-reservoir relationships, our results contribute towards establishing an experimental bat-filovirus pathogenesis model and interpretation of EBOV-seropositive results in wild-caught *R. aegyptiacus*. Understanding of the host mechanisms involved in abortive (following EBOV inoculation) *versus* systemic but sub-clinical infection (following Marburg virus inoculation) profiles in *R. aegyptiacus* might assist in a better understanding of filovirus pathogenesis.
